# The Impact of Ketogenic Capacity on Lipid Profile in Individuals with Prediabetes or Newly Diagnosed Type 2 Diabetes

**DOI:** 10.3390/ijms26178566

**Published:** 2025-09-03

**Authors:** Jaehyun Bae, Minyoung Lee, Yong-ho Lee, Sang-Guk Lee, Byung-Wan Lee

**Affiliations:** 1 Division of Endocrinology and Metabolism, Department of Internal Medicine, Hallym University Kangnam Sacred Heart Hospital, Hallym University College of Medicine, Seoul 07441, Republic of Korea; baejh0419@gmail.com; 2 Division of Endocrinology, Department of Internal Medicine, Yonsei University College of Medicine, Seoul 03722, Republic of Korea; lmycj@yuhs.ac (M.L.); yholee@yuhs.ac (Y.-h.L.); 3 Institute of Endocrine Research, Yonsei University College of Medicine, Seoul 03722, Republic of Korea; 4 Department of Laboratory Medicine, Yonsei University College of Medicine, Seoul 03722, Republic of Korea; comforter6@yuhs.ac

**Keywords:** ketogenesis, lipid, cardiovascular

## Abstract

In individuals with non-adipogenic traits and enhanced ketogenic capacity, plasma triglyceride (TG) levels are typically low, while low-density lipoprotein cholesterol (LDL-C) levels often exceed the normal range, complicating cardiovascular risk assessment. We analyzed lipid profiles to better characterize cardiovascular risk in this population. Drug-naïve patients newly diagnosed with prediabetes or type 2 diabetes (T2D) were divided into two groups based on serum β-hydroxybutyrate levels: enhanced versus non-enhanced ketogenesis. Among those with enhanced ketogenesis, 27 individuals with high LDL-C (≥100 mg/dL) and low TG (<150 mg/dL) were selected. For comparison, 27 individuals with high TG (>150 mg/dL) from the non-enhanced group were included. The enhanced ketogenesis group demonstrated more favorable lipid characteristics, including a significantly larger average LDL particle size (26.8 ± 0.3 nm vs. 25.9 ± 0.6 nm, *p* < 0.001), a lower proportion of small dense LDL particles, and reduced oxidized LDL to LDL-C ratio. Importantly, enhanced ketogenesis remained an independent predictor of larger LDL particle size after adjusting for potential confounders including TG. Despite the potential of selection bias intentionally induced by the predefined inclusion criteria, our findings suggest that patients with T2D or prediabetes who exhibit enhanced ketogenesis, even in the presence of elevated LDL-C levels, may have a more favorable atherogenic profile and are not necessarily at increased cardiovascular risk.

## 1. Introduction

During periods of low carbohydrate intake or starvation, ketone bodies including β-hydroxybutyrate (βHB), acetoacetate, and acetone are generated in the liver, from fatty acids, to serve as efficient fuels for peripheral organs [1]. Under physiological conditions, βHB levels are generally below 0.1 mmol/L, but they can rise to 0.5–5.0 mmol/L during fasting, ketogenic diets, or following aerobic exercise [2]. In patients with type 2 diabetes (T2D), mildly elevated βHB levels (0.1~1.5 mmol/L) are occasionally observed. Notably, a study of drug-naïve patients newly diagnosed with T2D reported a median βHB level of 0.1 mmol/L [3]. Weight loss effects of ketogenic diets and studies on the pleiotropic effects of sodium–glucose cotransporter-2 inhibitors have resulted in increasing interest in the beneficial effects of ketone bodies [4,5,6], not only as efficient fuels during fasting, but also as biomolecules with diverse physiological benefits.

Recent reports suggested that individual differences in ketogenic capacity, not induced by a certain diet or medication, are associated with metabolic outcomes. For instance, studies evaluating ketogenic capacity through urine or blood ketone measurements after overnight fasting have shown a lower risk of developing T2D and reduced severity of metabolic dysfunction-associated steatotic liver disease (MASLD) in individuals with adequate enhanced ketogenic capacity [7,8]. The lipid profiles of individuals with enhanced ketogenesis generally indicate low plasma triglyceride (TG) levels but similar or higher low-density lipoprotein-cholesterol (LDL-C) levels [3,9]. In addition, when lean individuals follow a ketogenic diet and exercise, their TG levels significantly decrease, while their LDL-C levels increase dramatically, a phenomenon known as the lean mass hyper-responder phenotype [10,11].

The tendency for increased LDL-C levels in individuals with enhanced or improved ketogenic capacity may cause ambiguity when assessing cardiovascular outcomes, as the accompanying low TG levels may reflect a fundamental difference from patterns observed in metabolically unhealthy individuals with elevated TG and LDL-C levels. Therefore, using a prospective cohort database of patients with prediabetes or newly diagnosed T2D and human-derived samples, we performed a detailed analysis of LDL subtypes and oxidation, as well as lipoprotein profiles, to explore the relationship between ketogenic capacity and lipid profiles.

## 2. Results

### 2.1. Clinical and Laboratory Characteristics of Participants

When comparing the lipid profiles of 166 patients from the prospective diabetes registry, the high βHB group tended to have lower TG levels (135.2 ± 64.4 vs. 159.3 ± 78.6 mg/dL; *p* = 0.033) and higher LDL-C levels (122.3 ± 44.0 vs. 108.8 ± 37.6 mg/dL; *p* = 0.035) (Table S1), consistent with findings from previous studies [3,9]. From an original cohort of 166 newly diagnosed patients with diabetes, 27 individuals with enhanced ketogenic properties and relevant lipid phenotype (defined as βHB ≥ 0.1 mmol/L, TG < 150 mg/dL, and ≥LDL-C 100 mg/dL) were selected, along with 27 individuals without enhanced ketogenesis (defined as βHB < 0.1 mmol/L and TG ≥ 150 mg/dL), as the control group.

The baseline characteristics of these 54 study participants are presented in Table 1. In the enhanced ketogenesis group, the proportion of males was significantly lower than that in the non-enhanced ketogenesis group (11 vs. 20, *p* = 0.027). There were no significant differences in age, body mass index (BMI), and glycated hemoglobin (HbA1c) between the two groups (48.2 ± 15.0 vs. 50.6 ± 12.2 years; *p* = 0.515, 26.4 ± 4.7 vs. 28.6 ± 5.1 kg/m^2^; *p* = 0.103, 7.5 ± 2.1 vs. 7.0 ± 1.2%; *p* = 0.229, respectively). In patients with enhanced ketogenesis, insulin sensitivity was significantly better when assessed using Homeostatic Model Assessment for Insulin Resistance (HOMA-IR, 3.4 ± 2.9 vs. 6.0 ± 5.2, *p* = 0.031).

As expected, due to the inclusion process, lipid profiles were significantly different between the groups. Patients with enhanced ketogenesis had lower plasma TG (110.1 ± 33.1 vs. 244.4 ± 73.6 mg/dL, *p* < 0.001) and higher LDL-C levels (133.1 ± 38.1 vs. 107.6 ± 30.8 mg/dL, *p* = 0.009) than those with non-enhanced ketogenesis. Plasma high-density lipoprotein-cholesterol (HDL-C) was higher in the enhanced ketogenesis group (58.5 ± 14.5 vs. 49.4 ± 14.6 mg/dL, *p* = 0.025).

The analysis of LDL subfractions revealed that mean LDL particle size was significantly larger in patients with enhanced ketogenesis (26.8 ± 0.3 vs. 25.9 ± 0.6 nm, *p* < 0.001), with a higher percentage of larger LDL1 and LDL2 particles and lower percentage of smaller LDL 3~7 particles (*p* < 0.001 for both measures).

Apolipoprotein A1 (ApoA1) levels were similar between the two groups, and variables associated with atherogenic lipoproteins, such as apolipoprotein B (apoB), lipoprotein (a) [lp(a)], and oxidized LDL levels, were not significantly different between the two groups. However, when adjusted for LDL-C levels, oxidized LDL was lower in the enhanced ketogenesis group (oxidized LDL to LDL-C ratio, 0.3 ± 0.1 vs. 0.6 ± 0.6, *p* = 0.039).

Serum levels of aspartate aminotransferase (AST), alanine aminotransferase (ALT), and total bilirubin, along with estimated glomerular filtration rate (eGFR), were statistically similar between the groups, indicating no significant liver or renal function discrepancies.

### 2.2. Mean LDL Particle Size Is Positively Correlated with Serum βHB Levels

We found that LDL particle size was significantly larger in patients with enhanced ketogenesis, although their LDL-C levels were higher. Consequently, we performed correlation analysis to identify variables associated with LDL particle size (Table 2). There was a significant positive correlation between serum βHB and mean LDL particle size (r = 0.595, *p* < 0.001) indicating a positive correlation between ketogenic capacity and LDL particle size.

In addition, BMI, hypertension, T2D, TG levels, and HOMA-IR negatively correlated with LDL particle size, whereas HDL-C and LDL-C levels positively correlated with LDL particle size. In terms of sex, females showed a positive correlation with LDL particle size.

### 2.3. Ketogenic Capacity Is Associated with Mean LDL Particle Size

In the unadjusted linear regression model, enhanced ketogenesis was a significant predictor, with a standardized coefficient β of 0.715 (*p* < 0.001), suggesting a strong positive association between enhanced ketogenesis and larger LDL particle size (Table 3). In the multivariable linear regression models, enhanced ketogenesis remained a strong significant predictor; after adjusting for age, sex, and BMI (β = 0.656, *p* < 0.001) in model 1, age, sex, BMI, and medical history including hypertension and T2D in model 2 (β = 0.633, *p* < 0.001), and age, sex, BMI, and metabolic variables including HOMA-IR, and TG in model 3 (β = 0.316, *p* = 0.027). Among the covariates, TG was significantly associated with mean LDL particle size, whereas age, sex, BMI, and HOMA-IR were not significant predictors in the multivariable models.

## 3. Discussion

Recently, there has been a shift from the conventional paradigm that high LDL-C levels are associated with a higher atherogenic tendency; elevated LDL-C levels induced by a ketogenic diet in healthy individuals are not associated with coronary plaques [12]. However, studies on the relationship between enhanced ketogenesis and lipid profiles in pathological conditions, such as diabetes, are lacking. To the best of our knowledge, this study is the first to suggest a relationship between ketogenic properties and lipid profiles in patients with dysglycemia, including those with prediabetes and newly diagnosed T2D, while excluding the potential confounding effects of antidiabetic and lipid-lowering drug use. Furthermore, in this study, we not only examined the blood lipid panel, but also measured LDL subtypes, oxidation status, and lipoprotein profiles, thereby obtaining detailed information regarding atherogenic properties. We identified comprehensive lipid profile characteristics of individuals with enhanced ketogenesis, defined by high βHB, low TG, and relatively high LDL-C levels, relative to appropriate comparators. Finally, we arrived at three main conclusions:

First, in patients with enhanced ketogenesis, even if LDL-C levels were high, the mean LDL particle size was larger, and the proportion of the LDL3–7 subfraction, referred to as small dense LDL (sdLDL), was significantly lower. SdLDL is considered to be a more atherogenic parameter than LDL-C concentration [13,14], because of its enhanced affinity for the arterial wall, increased penetration capability, and reduced hepatic clearance via LDL receptors [13]. This suggests that despite a higher blood LDL-C concentration, the risk of cardiovascular disease may not necessarily increase in patients with enhanced ketogenesis. In addition, the relatively lower proportion of sdLDL in the enhanced ketogenic group may explain why apoB, which particularly correlates with sdLDL, showed no difference between the comparison groups despite high LDL-C levels in the enhanced ketogenic group [14].

Second, similar levels of atherogenic lipoproteins, such as lp(a) and oxidized LDL, were observed in the enhanced and non-enhanced ketogenesis groups. Lp(a) is an LDL-like particle formed by the interaction of apolipoprotein(a) and apoB-100 [15], and is associated with cardiovascular disease independent of LDL-C or apoB [16]. Oxidized LDL is another well-known risk factor for cardiovascular disease [17]. Once LDL is oxidized, macrophages swallow the oxidized LDL and become foam cells, which accumulate in the arterial walls and gradually form atherosclerotic plaques [18]. Our results show that patients with enhanced ketogenesis and higher LDL-C levels had no significant differences in lp(a) and oxidized LDL levels compared to the control group, along with larger size of LDL particles, which supports the hypothesis that cardiovascular risk in these patients may not be markedly increased. In addition, a lower oxidized LDL-to-LDL ratio in the enhanced ketogenesis group may be considered a favorable factor for cardiovascular disease because a higher proportion of oxidized LDL to total LDL is an important risk factor for cardiovascular disease [19]. These findings highlight that the qualitative features of lipoproteins, rather than LDL-C concentration alone, may be more clinically relevant in assessing cardiovascular risk in patients with enhanced ketogenesis.

Third, in patients with enhanced ketogenesis, elevated LDL-C levels were commonly accompanied by higher HDL-C levels. Recent studies suggest that the protective effect of HDL-C on cardiovascular risk may exhibit a U-shaped association rather than a linear correlation [20,21]. However, because the HDL-C levels in our study were not extremely high or low, it can be interpreted in line with conventional understanding [22], that relatively high HDL-C levels may have a protective effect against cardiovascular risk. Therefore, considering that the LDL particle size is larger, atherogenic lipoproteins are not increased, and there is a tendency for higher HDL-C levels to act as a protective factor, it can be inferred that patients with enhanced ketogenesis may have a relatively favorable prognosis for cardiovascular disease, despite high LDL-C levels.

Among the three main findings described above, what we particularly noted is that individuals with enhanced ketogenesis and elevated LDL-C levels have significantly larger LDL particle sizes. Through regression analysis of LDL particle size, we identified ketogenic potency as a statistically significant predictor with a positive correlation with LDL particle size.

It is well-known that higher TG levels lead to the formation of sdLDL particles [23,24]. Our multiple regression analysis showed that TG was significantly associated with LDL particle size, as expected. As mentioned in the introduction, individuals with enhanced ketogenesis tend to have lower TG levels. Considering that the association between ketogenic potency and LDL particle size tended to decrease after adjusting for TG in the multiple regression analysis model, it can be inferred that the characteristics of large LDL particle size in individuals with enhanced ketogenesis are driven by mechanisms related to the reduction in TG levels. However, even after adjusting for TG, the association between ketogenic potency and LDL particle size remained statistically significant, suggesting that it acts as an independent predictor.

Individuals with enhanced ketogenesis can efficiently process fatty acids entering the liver through β-oxidation and ketogenic pathway, which helps to effectively control intrahepatic fat accumulation and de novo lipogenesis. This is supported by previous studies that showed that populations with enhanced ketogenesis had a lower incidence and severity of MASLD [8,9,25,26] and that ketogenesis was progressively impaired as hepatic steatosis worsened in patients with MASLD [27].

When the liver efficiently processes fatty acid influx and suppresses hepatic lipogenesis, it can reduce the secretion of very low-density lipoprotein (VLDL) [28,29], particularly TG-rich VLDL. Therefore, ketogenesis, the efficient and potent method for the disposal of fatty acids in the liver, is thought to suppress VLDL production. Additionally, in individuals with non-enhanced ketogenesis, a larger proportion of acetyl-CoA generated through β-oxidation enters the tricarboxylic acid (TCA) cycle instead of the ketogenic pathway [27]. The utilization of acetyl-CoA in the TCA cycle is associated with increased pyruvate carboxylase flux, which represents the anaplerotic pathway of the TCA cycle and is the control point for gluconeogenesis. Therefore, the activation of TCA cycle as an alternative pathway can cause hyperglycemia, which may also lead to an increase in hepatic de novo lipogenesis. This sequence of events can ultimately result in increased hepatic steatosis and elevated hepatic VLDL production, leading to elevated plasma TG levels and sdLDL formation.

Our study has several limitations. First, the small sample size and cross-sectional designs substantially limit the generalizability of our findings and preclude causal inference. In addition, participants were selected according to LDL-C and TG criteria, and therefore selection bias was inevitable. This approach was chosen in line with the aim of our study, which sought to examine the clinical significance of elevated LDL-C within the paradoxical context of low TG in ketogenic patients, through an in-depth analysis of lipid profiles. Although this selection process allowed for the possibility of baseline imbalance, apart from the intended differences in lipid profiles there were no statistically significant between-group differences in other parameters including BMI, glucose levels, liver enzymes, and eGFR. However, the resulting selection bias, combined with the limited sample size, should be regarded as one of the major statistical constraints of our study. Nevertheless, we believe that our findings provide an important clinical perspective on the phenomenon of low TG and high LDL-C observed under conditions of enhanced ketogenesis, and may serve as a starting point for larger, prospective investigations in the future. Second, our study lacked direct mechanistic insights into the association between ketogenesis and characteristic lipid profiles. Finally, we did not control for lifestyle factors such as diet and physical activity, which could have confounded our results. However, to minimize confounding factors, we only included prediabetic or newly diagnosed patients with T2D who had no history of using antidiabetic or lipid-lowering medications.

In conclusion, our findings suggest that among drug-naïve patients with prediabetes or T2D, those with enhanced ketogenesis showed larger LDL particle size, comparable levels of other atherogenic lipoproteins such as oxidized LDL and lp(a), and higher HDL-C levels, even in the presence of elevated LDL-C. This trend is expected to be more pronounced in individuals with enhanced ketogenesis and relatively low TG levels. Given the cross-sectional design and small sample size, these results should be interpreted with caution. Nevertheless, we consider our findings to serve as a proof-of-concept study that may stimulate larger prospective investigations, such as those incorporating dietary or exercise interventions to actively induce ketogenesis and longitudinally evaluate its impact on lipid profiles and clinical outcomes.

## 4. Materials and Methods

### 4.1. Study Design and Population

In this cross-sectional study, 166 participants were initially recruited from a prospective diabetes registry at the Severance Diabetes Center, Seoul, Korea, between March 2022 and July 2024. The diabetes registry enrolled individuals aged ≥ 20 years with prediabetes and T2D, with no prior history of taking antidiabetic drugs. The diabetes registry comprised individuals who underwent a standardized mixed-meal stimulation test during their first visit to a diabetes center. The registry protocol included routine collection of blood samples at 0 and 90 min (basal and stimulated, respectively) to analyze glucose, insulin, and C-peptide levels.

Residual blood samples (1.5 mL) from routine blood chemistry tests were collected only from participants who provided consent at the time of registry enrollment. These blood samples were aliquoted into smaller portions (300 μL) and stored at −70 °C for further analysis.

The inclusion criteria for this study were as follows: prediabetes or newly diagnosed with T2D based on the 2023 guidelines of the Korean Diabetes Association [30], with available baseline βHB levels, lipid profile results, and residual blood samples. Patients who had received organ transplantation or chemotherapy, those who used steroids, had previously taken antidiabetic or lipid-lowering drugs before the initial blood sampling, or visited the emergency room due to hyperglycemia were excluded. We further excluded individuals with insufficient remaining blood samples, as well as those with TG > 500 mg/dL, missing or unmeasurable LDL-C values.

After applying the inclusion and exclusion criteria, participants were classified into two groups based on serum βHB levels: those with βHB ≥ 0.1 mmol/L were categorized as enhanced ketogenesis group and those with βHB < 0.1 mmol/L were categorized as non-enhanced ketogenesis group. Applying 0.1 mmol/L as the cutoff is supported by prior evidence: as described in the Introduction, βHB levels in healthy individuals generally do not exceed 0.1 mmol/L, and in a study of patients with T2D, the median βHB level was likewise reported to be 0.1 mmol/L [2,3].

To highlight the lipid characteristics of individuals with enhanced ketogenic capacity, we defined the enhanced ketogenesis group as 27 participants who met the criteria of βHB ≥ 0.1 mmol/L, TG < 150 mg/dL, and LDL-C ≥ 100 mg/dL, in accordance with the clinical practice guidelines [30,31]. For comparison, an equal number of individuals with βHB < 0.1 mmol/L and TG > 150 mg/dL were selected from the non-enhanced ketogenesis group. The sample size of each study group was determined using the G*Power 3.1.9.7 program to achieve 90% power (two-sided, α = 0.05). The calculated minimal sample size for the study was at least 5 to 26 participants in each group, based on the mean difference and standard deviation observed in a previous study testing the impact of metabolic status on LDL particle size and lipid proteins [32,33].

The study protocol was approved by the Institutional Review Board of Severance Hospital (4–2022–0234 and 4–2024–1435) and Kangnam Sacred Heart Hospital (2024–10–003). All participants provided written informed consent for the diabetes registry study (4–2022–0234), and the requirement for informed consent was waived for the current cross-sectional study (4–2024–1435) because of its retrospective nature.

### 4.2. Clinical Measurement and Laboratory Assessment

Patient demographics including age, sex, BMI, medication history, and blood chemistry and clinical and biochemical measurements at the time of screening were obtained by reviewing electronic medical records. The following laboratory variables were measured at screening as baseline variables after overnight fast: serum βHB, glucose, HbA1c, insulin, C-peptide, total cholesterol, TG, HDL-C, LDL-C, AST, ALT, total bilirubin, and creatinine. BMI was calculated as body weight divided by height squared (kg/m^2^), and eGFR was assessed using the Chronic Kidney Disease Epidemiology Collaboration (CKD-EPI) equation. The homeostasis model assessments of insulin resistance, and β-cell function (HOMA-IR, and HOMA-β) were calculated to assess insulin resistance and pancreatic β-cell function [34,35].

Using blood samples stored in a frozen state, we first remeasured total cholesterol, TG, HDL-C, and LDL-C levels and used them in our analysis. The LDL subfraction profile was investigated using polyacrylamide gel tube electrophoresis (Lipoprint System; Quantimetrix, Redondo Beach, CA, USA), and data on mean LDL particle size and distribution of seven LDL-C subclasses, LDL1 and LDL2 (large buoyant subfractions), and LDL3–7 (small atherogenic subfractions), was obtained. In addition, apoB and lp(a) were measured by an immunoturbidimetric assay using the Cobas 8000 instrument c702 module (Roche, Mannheim, Germany). Oxidized LDL, which are atherogenic lipoproteins, was measured using an enzyme-linked immunosorbent assay method (Mercodia, Uppsala, Sweden), and apoA1, a protective factor, were also measured using an immunoturbidimetric method (Roche). Finally, the frozen-stored blood samples were transported to SCL healthcare laboratory (Seoul Clinical Laboratories, Yongin, Republic of Korea) to measure the concentration of serum acetoacetate and βHB, forms of the ketone bodies, using gas chromatography–mass spectrometry. This process was performed because institution did not measure serum acetoacetate, and βHB could not be quantified at concentrations < 0.1 mmol/L with our instrument.

### 4.3. Statistical Analysis

The characteristics of the study participants were analyzed according to the status of ketogenesis using Student’s t-test for continuous variables and χ^2^ test for categorical variables. All continuous variables are presented as the mean ± standard deviation, and categorical variables are presented as percentage (%). Correlations between mean LDL particle size and other variables were assessed using Spearman’s correlation coefficient. Multivariate linear regression analysis was used to determine the independent association between ketogenic potency and mean LDL particle size. Statistical analyses were performed using SPSS version 28.0 for Windows (IBM Corp., Armonk, NY, USA). Statistical significance was set at *p* < 0.05.

## Figures and Tables

**Table 1:** Characteristics of the study population newly diagnosed with prediabetes or type 2 diabetes according to ketogenic capacity.

Variables	Enhanced Ketogenesis (N = 27)	Non-Enhanced Ketogenesis (N = 27)	*p*-Value
Age (years)	48.2 ± 15.0	50.6 ± 12.2	0.515
Sex [Male, n (%)]	11 (40.7)	20 (74.1)	0.027
BMI (kg/m^2^)	26.4 ± 4.7	28.6 ± 5.1	0.103
Hypertension [n (%)]	3 (11.1)	24 (88.9)	0.003
Type 2 diabetes [n (%)]	16 (59.3)	11 (40.7)	0.248
βHB (mmol/L)	0.20 ± 0.2	0.03 ± 0.02	<0.001
Acetoacetate (mmol/L)	0.04 ± 0.04	0.01 ± 0.009	<0.001
Fasting glucose (mg/dL)	134.3 ± 62.2	129.4 ± 38.6	0.730
Postprandial glucose (mg/dL)	178.1 ± 85.6	179.0 ± 58.8	0.963
HbA1c (%)	7.5 ± 2.1	7.0 ± 1.2	0.229
Fasting insulin (μIU/mL)	9.6 ± 6.5	18.0 ± 15.3	0.013
Fasting c-peptide (μIU/mL)	2.2 ± 1.0	3.6 ± 1.4	<0.001
Postprandial insulin (μIU/mL)	47.1 ± 32.2	73.1 ± 47.9	0.027
Postprandial c-peptide (μIU/mL)	5.7 ± 2.5	7.2 ± 2.3	0.024
HOMA-IR	3.4 ± 2.9	6.0 ± 5.2	0.031
HOMA-β	62.4 ± 43.2	118.8 ± 133.0	0.052
AST (IU/L)	35.4 ±35.3	42.6 ±32.9	0.442
ALT (IU/L)	44.4 ± 37.7	58.2 ± 46.3	0.236
Total bilirubin (mg/dL)	0.9 ± 0.4	0.8 ± 0.2	0.158
eGFR (ml/min/1.73 m^2^)	95.6 ± 18.8	94.3 ± 16.6	0.781
Total cholesterol (mg/dL)	210.0 ± 32.2	195.6 ± 34.4	0.117
TG (mg/dL)	110.1 ± 33.1	244.4 ± 73.6	<0.001
HDL-C (mg/dL)	58.5 ± 14.5	49.4 ± 14.6	0.025
LDL-C (mg/dL)	133.1 ± 38.1	107.6 ± 30.8	0.009
Mean LDL particle size (nm)	26.8 ± 0.3	25.9 ± 0.6	<0.001
LDL1,2 (%)	37.0 ± 4.5	24.3 ± 6.4	<0.001
LDL3~7 (%)	3.8 ± 3.0	11.3 ± 5.6	<0.001
Apolipoprotein A1 (mg/dL)	161.0 ± 34.2	156.0 ± 37.9	0.616
Apolipoprotein B (mg/dL)	116.0 ± 21.7	114.3 ± 24.5	0.791
Lipoprotein (a) (mg/dL)	16.1 ± 14.8	14.4 ± 16.6	0.690
Oxidized LDL (U/L)	48.2 ± 15.0	47.2 ± 9.0	0.762
Oxidized LDL to LDL ratio	0.3 ± 0.1	0.6 ± 0.6	0.039

Continuous variables expressed as means ± standard deviation (SD); categorical variables expressed as number (percent). *p* < 0.05 denotes statistical significance. Abbreviations: BMI, body mass index; βHB, β-hydroxybutyrate; HbA1c, glycated hemoglobin; HOMA-IR, homeostasis model assessments of insulin resistance; HOMA-β, homeostasis model assessments of β-cell function; AST, aspartate aminotransferase; ALT, alanine aminotransferase; eGFR, estimated glomerular filtration rate; TG, triglyceride; HDL-C, high-density lipoprotein-cholesterol; LDL-C, low-density lipoprotein-cholesterol.

**Table 2:** Simple correlation analysis to find factors correlated with the mean LDL particle size.

Variables	r	*p*-Value
Age (years)	0.054	0.696
Sex (female vs. male)	0.437	<0.001
BMI (kg/m^2^)	−0.324	0.017
Hypertension (yes vs. no)	−0.323	0.017
Type 2 diabetes (vs. prediabetes)	−0.296	0.030
βHB (mmol/L)	0.595	<0.001
HOMA-IR (mg/dL × μIU/mL)	−0.325	0.020
HOMA- β (%)	−0.131	0.359
TG (mg/dL)	−0.810	<0.001
HDL-C (mg/dL)	0.582	<0.001
LDL-C (mg/dL)	0.495	<0.001
HbA1c (%)	−0.082	0.557

Abbreviations: BMI, body mass index; βHB, β-hydroxybutyrate; HOMA-IR, homeostasis model assessments of insulin resistance; HOMA-β, homeostasis model assessments of β-cell function; TG, triglyceride; HDL-C, high-density lipoprotein-cholesterol; LDL-C, low-density lipoprotein-cholesterol; HbA1c, glycated hemoglobin.

**Table 3:** Linear regression model for the mean LDL particle size.

	R^2^	Standardized Coefficient β	*p*-Value
Unadjusted	0.512		
Enhanced ketogenesis (βHB ≥ 0.1 mmol/L vs. <0.1 mmol/L)		0.715	<0.001
Model 1	0.545		
Age (years)		0.039	0.713
Sex (female vs. male)		0.172	0.110
BMI (kg/m^2^)		−0.022	0.840
Enhanced ketogenesis (βHB ≥ 0.1 mmol/L vs. <0.1 mmol/L)		0.656	<0.001
Model 2	0.571		
Age (years)		0.023	0.836
Sex (female vs. male)		0.172	0.108
BMI (kg/m^2^)		0.007	0.949
Hypertension (yes vs. no)		−0.004	0.969
Type 2 diabetes (vs. prediabetes)		−0.168	0.112
Enhanced ketogenesis (βHB ≥ 0.1 mmol/L vs. <0.1 mmol/L)		0.633	<0.001
Model 3	0.655		
Age (years)		0.045	0.647
Sex (female vs. male)		0.156	0.093
BMI (kg/m^2^)		0.023	0.824
HOMA-IR (mg/dL × μIU/mL)		0.027	0.807
TG (mg/dL)		−0.514	<0.001
Enhanced ketogenesis (βHB ≥ 0.1 mmol/L vs. <0.1 mmol/L)		0.316	0.027

Abbreviations: BMI, body mass index; HOMA-IR, homeostasis model assessments of insulin resistance; TG, triglyceride.

## Data Availability

Data pertaining to the manuscript will be shared upon reasonable request to Byung-Wan Lee (Yonsei University College of Medicine, bwanlee@yuhs.ac).

## Supplementary Materials

The following supporting information can be downloaded at: https://www.mdpi.com/article/10.3390/ijms26178566/s1.
