# Supplementary material for: The Impact of Ketogenic Capacity on Lipid Profile in Individuals with Prediabetes or Newly Diagnosed Type 2 Diabetes
Source: Int J Mol Sci. 2025 Sep 3;26(17):8566. doi: 10.3390/ijms26178566 (PMC12429389; doi:10.3390/ijms26178566)
Supplement: Supplementary file 1 [file ijms-26-08566-s001.zip › ijms-3799286-supplementary.pdf]

Table S1. Characteristics of the initially recruited study population newly diagnosed with prediabetes or type 2 diabetes according to ketogenic capacity

Variables	Enhanced ketogenesis* (N=82)	Non-enhanced ketogenesis (N=84)	P-value
Age (years)	50.8 ± 14.8	53.3 ± 13.2	0.257
Sex [Male, n (%)]	44 (53.7)	57 (67.9)	0.061
BMI (kg/m^2)	27.0 ± 4.6	27.8 ± 4.4	0.216
Hypertension [n (%)]	19 (23.2)	37 (44.0)	0.004
Type 2 diabetes [n (%)]	59 (72.0)	62 (73.8)	0.788
Dyslipidemia [n (%)]	25 (30.5)	33 (39.3)	0.235
Fasting glucose (mg/dL)	154.5 ± 67.9	128.9 ± 33.8	0.003
Postprandial glucose (mg/dL)	186.4 ± 73.2	183.1 ± 56.7	0.776
HbA1c (%)	8.4 ± 2.4	7.1 ± 1.3	<0.001
Fasting insulin (μIU/mL)	11.7 ± 10.1	16.7 ± 14.8	0.016
Fasting c-peptide (μIU/mL)	2.5 ± 1.1	3.2 ± 1.7	0.004
Postprandial insulin (μIU/mL)	48.2 ± 32.3	65.1 ± 43.3	0.008
Postprandial c-peptide (μIU/mL)	5.7 ± 2.5	6.7 ± 2.3	0.020
HOMA-IR	4.7 ± 4.8	5.2 ± 4.7	0.517
HOMA- β	62.6 ± 56.8	117.4 ± 125.5	<0.001
AST (IU/L)	34.0 ± 25.5	36.3 ± 24.2	0.547
ALT (IU/L)	41.5 ± 37.9	44.4 ± 32.6	0.593
Total bilirubin (mg/dL)	0.9 ± 0.4	0.8 ± 0.3	0.030
eGFR (ml/min/1.73m^2)	92.4 ± 15.8	91.5 ± 17.4	0.736
Total cholesterol (mg/dL)	199.8 ± 45.5	186.7 ± 39.6	0.049
TG (mg/dL)	135.2 ± 64.4	159.3 ± 78.6	0.033
HDL-C (mg/dL)	48.9 ± 11.9	46.9 ± 12.4	0.271
LDL-C (mg/dL)	122.3 ± 44.0	108.8 ± 37.6	0.035

*Enhanced ketogenesis was defined as βHB ≥ 0.1 mmol/L, and non-enhanced ketogenesis as βHB < 0.1 mmol/L.

Continuous variables expressed as means ± standard deviation (SD); categorical variables expressed as number (percent). $P < 0.05$ denotes statistical significance.
Abbreviations: βHB, β-hydroxybutyrate, BMI, body mass index; AST, aspartate aminotransferase; ALT, alanine aminotransferase; eGFR, estimated glomerular filtration rate; TG, triglyceride; HDL-C, high density lipoprotein-cholesterol; LDL-C, low density lipoprotein-cholesterol
